# Flavonol Glycosides from *Euphorbia microsciadia* Bioss. with their Immunomodulatory Activities

**Published:** 2012

**Authors:** Syed Mustafa Ghanadian, Abdul Majid Ayatollahi, Suleiman Afsharypour, Sumaira Hareem, Omer Mohamed Abdalla, Jean Jules Kezetas Bankeu

**Affiliations:** a Isfahan Pharmaceutical Sciences Research Center, Isfahan University of Medical Sciences, Isfahan, Iran.; b School of Pharmacy andPhytochemistry Research Centre, Shahid Beheshti University of Medical Sciences, Tehran, Iran.; c School of Health. eFaculty of Pharmacy and Pharmaceutical Sciences, Isfahan University of Medical Sciences, Isfahan, Iran.; d Dr. Panjwani Center for Molecular Medicine and Drug Research, Sciences, University of Karachi, Karachi-75270, Pakistan.; e Department of Organic Chemistry, Faculty of Science, TWAS Research Unit of University of Yaounde I; Yaounde, Cameroon.

**Keywords:** *Euphorbia microsciadia*, Flavonoids, Immunomodulatory activity, Flavonol glycosides

## Abstract

Four known flavonoids: quercetin 3-*O*-*β*-D-rutinoside (Q3Rut), myricetin 3-*O*-*β*-D-galactopyranoside (M3Gal), quercetin 3-*O*-*β*-D-galactopyranoside (Q3Gal) and quercetin 3-*O*-*β*-D-glucopyranoside (Q3Glc), for the first time were isolated from aerial parts of *Euphorbia microsciadia*. The chemical structure of them was elucidated on the basis of 1 and 2 D-NMR spectra and different spectroscopic techniques. The immunomodulatory activities of isolated compounds were compared using standard T-cell proliferation assay. These data showed that lymphocyte suppression activity of flavonoids (1-4) were comparatively lower than prednisolon as a standard drug. Immunosuppressive activity of flavonoids with hydroxyl groups at both 3′-and 4′-positions in their B-ring (Q3Gal) were more than those with 3′-,4′-and 5′-hydroxyl substitution (M3Gal). In these compounds, Q3Gal showed the most inhibitory activity, whereas M3Gal showed the least lymphocyte antiprolifeartive activity.

## Introduction

**Figure 1:**
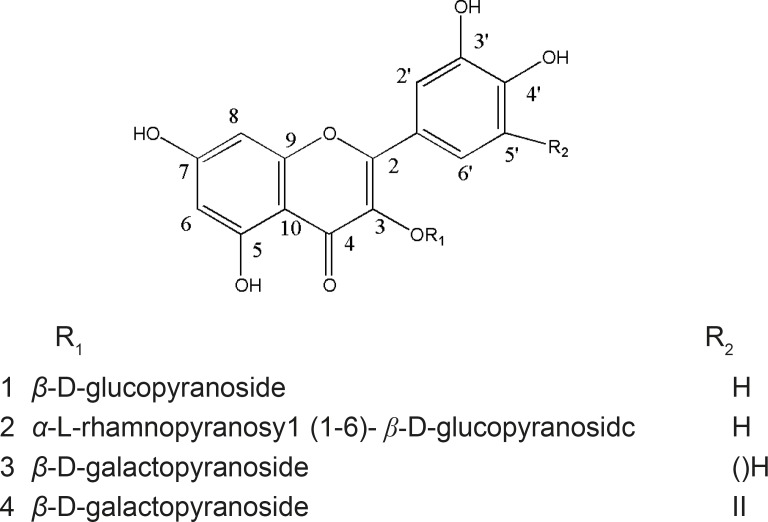
Flavonol glycosides (compounds 1-4) from *Euphorbia microsciadia*

*Euphorbia microciadia*, belonging to the family Euphorbiaceae, is a native herb grows in Iran (1). The previous studies on methanolic extract of the genus resulted in cytotoxic and immunomodulatory properties (2). Traditionally, *Euphorbia *is used in folk medicine as treatment of inflammations and tumors (3). In this paper, we reported the isolation, identification and biological activities of five flavonol glycosides (Figure 1) from the aerial parts of this plant. Although flavonoids are not considered as nutrients, they are important components of the human diet. Flavonoids have been reported to have many biological effects as antioxidant, anti-inflammatory, anticancer, and anti-ischemic. Moreover, they are able to inhibit protein-tyrosine kinase and protein kinase C (PKC) during lymphocyte activation (4-6). Therefore, in this study we investigated the human lymphocyte proliferation inhibitory effect of the isolated flavonol glycosides, using standard lymphocyte proliferation assay to evaluate their activity for the control of harmful immune responses.

## Experimental

*General*

Isolation of compounds were done with reverse column chromatography using RP-18 (40–63 μm, Merck, Germany), and recycling preparative HPLC equipped with UV and RI detectors (LC-908, Hitachi, Japan) using a YMC-M-18 column (250 × 20 mm i.d., YMC, Japan). The structures of compounds were elucidated using spectral methods ^1^H NMR, APT, COSY, HMBC, FT-IR, UV–vis and HR-ESIMS. The NMR spectra were acquired with Bruker Avance. The Infra red spectra were obtained by JASCO 302-A spectrophotometer, with KBr discs. The HRESI-MS spectra were acquired with Waters Q-TOF Micro YA019 mass spectrometer in *m/z *and EI-MS spectra with Varian MAT 112 or MAT 312 spectrometers. 

*Plant material*

Aerial flowering parts of *Euphorbia microsciadia *(Euphorbiaceae) was collected from Ghalil-e-Shirvan, Northern Khorasan province (Iran). Plant material was identified by Y. Naseh, plant taxonomist and a voucher specimen (nos. 2023) deposited in the herbarium of the Faculty of Pharmacy, and Pharmaceutical Sciences at the Isfahan University of Medical Sciences (Iran). 

*Extraction and isolation*

The air-dried powder of the plant material (4.5 kg) was soaked in methanol (20 L × 3) at room temperature for two weeks, and the resulting extract was concentrated to a green gummy residue. Methanolic extract was dissolved in distilled water and defatted with pet. ether. The defatted aqueous extract was further fractionated with EtOAc and BuOH. The butanolic fractions were subjected on MPLC (RP18; H_2_O/ MeCN, 5→50) to afford four fractions: F1–4. TLC Scanner analysis showed that F3 contained flavonoids. Therefore, F3 eluted with H_2_O/MeCN (7:3), was chromatographed on RP-18 CC (H_2_O/ MeCN, 7:3) to render several fractions: F3a-h. Then, F3b, contained mixtures of flavonoids and pigments, was further separated on another RP-18 CC (H_2_O/MeCN, 7:3) to yield F3b1-F3b7. Then, F3b4, and F3b5 each about 50 mg was purified by polyamide SC6 (DCM/MeOH, 8→20)*. *Fractions were purified more by recycling HPLC using columns M-18 (MeOH/H_2_O, 4:6). Finally compounds 1 (11mg)**, **2 (20 mg), 3 (8 mg), and 4 (20 mg) were obtained as pure compound. 

*Quercetin 3-O-β-D-glucopyranoside (compound 1)*

Pale yellow powder. ^1^H-NMR (500 MHz, MeOD) δ 3. 22 (ddd, J = 9, 5. 6, 2. 5 Hz, H-5″), 3. 34 (d, J = 9 Hz, H-4››), 3. 41 (t, J = 9 Hz, H-3″), 3. 47 (dd, J = 9, 7. 5 Hz, H-2″), 3. 56 (dd, J = 12, 5. 6 Hz, H-6a″), 3. 70 (dd, J = 12, 2. 5 Hz, H-6b″), 5. 15 (d, J = 7. 5 Hz, H-1″), 6. 11 (d, J = 2. 0 Hz, H-6), 6. 28 (d, J = 2. 0 Hz, H-8), 6. 84 (d, J = 8. 5 Hz, H-5′), 7. 57 (dd, J = 8. 5, 2 Hz, H-6′), 7. 70 (d, J = 2 Hz, H-2′). 13C-NMR (125 MHz, MeOD) δ 62. 5 (C-6″), 71. 2 (C-4″), 75. 7 (C-2″), 78. 1 (C-3″), 78. 3 (C-5″), 94. 8 (C-8), 100. 0 (C-6), 105. 3 (C-1″), 105. 6 (C-10), 117. 7 (C-2′), 116. 1 (C-5′), 122. 9 (C-1′), 123. 1 (C-6′), 135. 7 (C-3), 145.8 (C-3′), 150. 6 (C-4′), 158. 8 (C-2), 158. 4 (C-9), 163. 1 (C-5), 166. 3 (C-7), 179. 5 (C-4). Positive HR-FABMS m/z 465.1070 (calcd. for C_21_H_20_O_12_ + H^+^, 465. 1027); EI-MS *m/z *358, 326, 302, 273, 245, 229, 173, 153, 150, 144, 137, 133, 122, 105, 85, 77, 69, 60.

*quercetin 3-O-α-L-rhamnopyranosyl (1→6)- β -D-glucopyranoside (compound 2)*

Pale Yellow powder; ^1^H NMR (500 MHz, DMSO) *δ *1. 0 (3H, *d, J *= 6 Hz, H-6′′′), 3. 06 (m, H-3′′′), 3. 07 (m, H-4′′′), 3. 22 (m, H-3″), 3. 22 (m, H-2′′), 3. 25 (m, H-5′′), 3. 27 (m, H-4′′), 3. 28 (m, H-5′′′), 3. 29 (m, H-6′′′), 3. 39 (m, H-2′′′), 3. 71 (d, *J *= 10. 5, H-6″), 4. 39 (d*, J *= 1Hz, H-1″′), 5. 35 (*d, J *= 7. 5 Hz, H-1″), 6. 19 (d*, J *= 2 Hz, H-6), 6. 39 (d*, J *= 2 Hz, H-8), 6. 84 (d, *J *= 8 Hz, H-5′), 7. 54 (*br s, *H-2′), 7. 55 (dd*, J *= 8, 2. 5 Hz, H-6′); ^13^C NMR (125 MHz, DMSO) δ 17. 8 (C-6″′), 66. 9 (C-6″), 68. 2 (C-5″′), 69. 9 (C-3″′), 70. 3 (C-2″′), 70. 4 (C-4″), 71. 7 (C-4″′), 73. 9 (C-2″), 75. 8 (C-5″), 76. 3 (C-3″), 100. 6 (C-1″′), 101. 1 (C-1″), 93. 4 (C-8), 98. 5 (C-6), 103. 7 (C-10), 115. 0 (C-2′), 116. 0 (C-5′), 120. 9 (C-1′), 121. 3 (C-6′), 129. 9 (C-3), 144. 4 (C-3′), 148. 1 (C-4′), 156. 1 (C-2), 160. 9 (C-5), 163. 8 (C-7) , 177. 0 (C-4). Positive HR-FAB *m/z *611.1648 (calcd. for C_27_H_30_O_16_ +H^+^, 611. 1606, Δ 6. 9 ppm). ESI-MS *m/z *611 (M), 465 (M-Rha), 303 (M-Rha-Glc); EI-MS: 302, 273, 245, 228, 200, 153, 150, 137, 109, 108, 91, 81, 71, 69, 66. 

*Myricetin 3-O-β-D-galactopyranoside (compound 3)*

Pale yellow powder. 1H-NMR (400 MHz, DMSO) δ 3. 26 (m, H-6″), 3. 29 (m, H-5″ ), 3. 37 (dd, J = 3, 9. 2 Hz, H-3″ ), 3. 47 (dd, J = 14, 9. 6 Hz, H-6b″ ), 3. 61 (dd, J = 7. 6, 9. 2 Hz, H-2″), 3. 65 (d, J = 3 Hz, H-4″), 5. 29 (d, J = 7. 6 Hz, H-1″), 6. 18 (d, J = 1. 6 Hz, H-6), 6. 37 (2H, d, J = 1. 6 Hz, H-8), 7. 19 (2H, s, H-2′, H-6′), 8. 00 (OH), 12. 58 (5-OH). 13C-NMR (125 MHz, DMSO) δ 60. 1 (C-6″), 67. 9 (C-4″), 71. 2 (C-2″), 73. 2 (C-3″), 75. 9 (C-5″), 93. 3 (C-8), 98. 6 (C-6), 102. 0 (C-1″), 105. 8 (C-10), 108. 6 (C-2″, C-6″), 120. 0 (C-1′), 133. 7 (C-3), 136. 8 (C-4′), 145. 2 (C-3′, C-5′), 156. 2 (C-2, C-9), 161. 2 (C-5), 164. 3 (C-7), 177. 6 (C-4). Positive HR-FABMS *m/z *481.0996 (calcd. for C_21_H_20_O_13_ + H^+^, 481. 0976); EI-MS *m/z *302, 286, 231, 201, 164, 153, 149,126, 105, 95, 84, 73, 66. 

*Quercetin 3-O-β-D-galactopyranoside (compound 4)*

Pale yellow powder. 1H-NMR (500 MHz, MeOD) δ 3. 46 (dd, J = 6. 5, 6 Hz, H-5′′), 3. 53 (m, H-3′′), 3. 56 (m, H-6a′′), 3. 63 (dd, J = 11. 5, 6 Hz, H-6b′′), 3. 81 (dd, J = 9. 5, 7. 5 Hz, H-2′′), 3. 84 (br-d, J = 3 Hz, H-4′′), 5. 12 (d, J = 7. 5 Hz, H-1′′), 6. 16 (d, J = 2. 5 Hz, H-6), 6. 35 (d, J = 2. 0 Hz, H-8), 6. 85 (d, J = 8. 5 Hz, H-5′), 7. 57 (dd, J = 8. 5, 2 Hz, H-6′), 7. 82 (d, J = 2 Hz, H-2′). 13C-NMR (150 MHz, MeOD) δ 61. 9 (C-6′′), 69. 8 (C-4′′), 73. 4 (C-2′′), 75. 0 (C-3′′), 77. 2 (C-5′′), 95. 2 (C-8), 100. 5 (C-6), 105. 2 (C-1′′), 105. 5 (C-10), 117. 3 (C-2′), 116. 0 (C-5′), 122. 8 (C-1′), 123. 1 (C-6′), 135. 6 (C-3), 145. 9 (C-3′), 150. 8 (C-4′), 158. 4 (C-2), 158. 4 (C-9), 163. 0 (C-5), 169. 0 (C-7), 179. 5 (C-4). Positive HR-FABMS *m/z *465.1085 (calcd. for C_21_H_20_O_12_ + H^+^, 465. 1027); EI-MS *m/z *302, 273, 245, 229, 173, 153, 150, 144, 137, 133, 85, 60.

*Lymphocyte proliferation assay*

Peripheral human blood lymphocytes were incubated with different concentrations of the test compounds (0. 5, 5, and 50 μg/mL) in triplicates in supplemented RPMI-1640 along with phytohemagglutinin (PHA) at 37º C in CO_2_ environment for 72 h. Further incubation for 18 hours after the addition of thymidine (^3^H) (Amersham, Buckinghamshire, UK) was done and cells were harvested using cell harvester (Inotech Dottikon, Switzerland). Finally, proliferation level was determined by the radioactivity count as CPM reading recorded from *β*-scintillation counter (Beckman Coulter, LS 6500, Fullerton, CA, USA) (7).

*Statistical analysis*

All data are reported as mean ± SD of the mean and the IC_50_ values were calculated using Excel based program. One-way ANOVA: post-hoc dunnett test was also used and * p < 0.05; ** p < 0.01; *** p < 0.001 were considered to indicate a statistically significant difference.

## Results and Discussion

**Figure 2:**
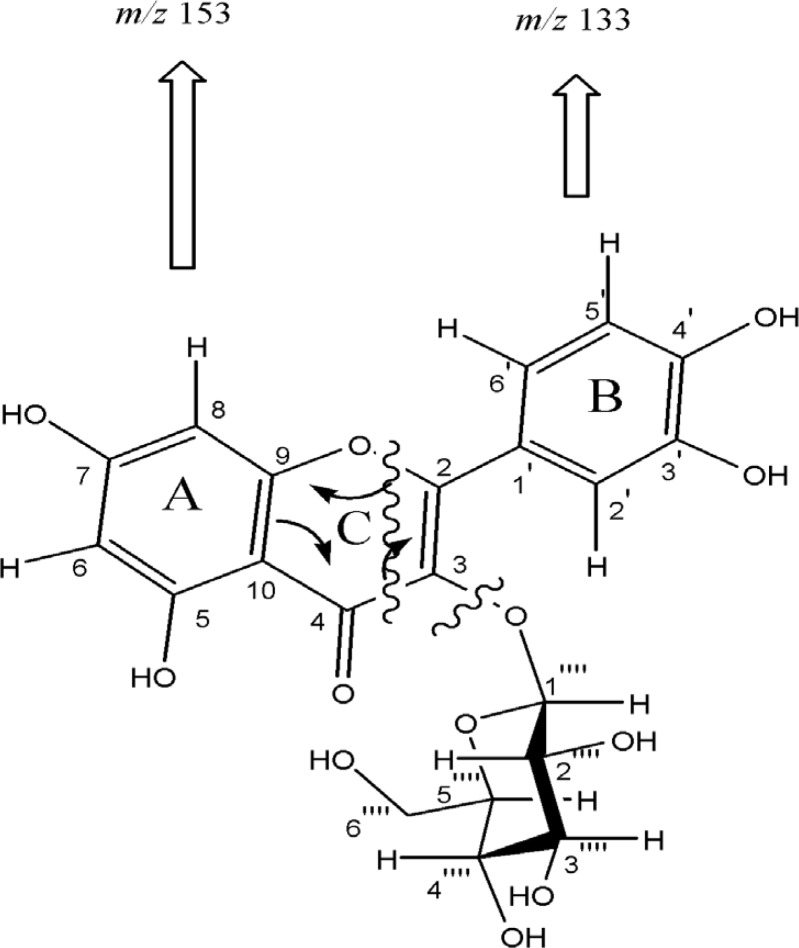
EI-Mass fragmentation pattern of compound 1. Cleavage of ring C by retro-Diels-Alder mechanism led to A (m/z 153) and B (m/z 133) ions

Compound 1, was obtained as pale yellow solid with positive reaction to methanolic ferric chloride and natural product reagent. The twelve degrees of unsaturation derived from positive HR-FABMS *m/z *465.1070 (calcd. for C_21_H_20_O_12_ + H^+^, 465. 1027), and NMR data, suggested presence of one carbonyl carbons, seven olefinic bands, and therefore, four rings in the skeleton. The UV spectrum showed absorption maxima at 270 and 350 nm, characteristic of flavone derivatives (8). The NMR spectrum of the aglycone moieties proved the presence of one carbonyl carbon resonances at δ_C_ 179. 5 (C-4), together with IR absorption at 3422 (OH) and 1654 cm^–1^ (conjugated C=O) indicating the presence of chelated phenolic hydroxyl group at the C-5 positions (9,10). The^ 1^H-NMR spectra displayed a signal at δ 12. 6 for a chelated hydroxyl group and *meta *coupled doublets at δ 6. 11 (1H, d, *J *= 2 Hz) and 6. 28 (1H, d, *J *= 2 Hz) described to H-6 and H-8. In addition, *ortho *coupled proton signals at δ 6. 84 (1H, d, *J *= 8. 5 Hz) and 7. 57 (1H, dd, *J *= 8.5, 2 Hz) corresponding to H- 5’ and H-6′ as well as δ 7. 70 (1H, d, J = 2 Hz) corresponding to H-2′ proton, indicated that compound 1 is a flavonol derivative. After acid hydrolysis of 1, the aqueous layer was separated and checked by TLC. Co-TLC with standard sugars and the assignments (chemical shifts, multiplicities, and coupling constants) ascribed sugar as glucopyranosyl (11, 12). The glycosyl linkage at C-3, was observed through C-H long-range correlations between anomeric proton H-1′′ (*δ*_H_ 5. 23) and carbon C-3 (*δ*_C_ 135. 7). The configurations of anomeric proton was deduced to be *β*-form based on coupling constants (H-1′′, *J *= 7.5 Hz). Regarding above considerations, and the similarity of the NMR data with similar structures, compound 1 is most probably Quercetin 3-O-*β*-D-glucopyranoside (13). It was further confirmed by analyzing EI mass spectrum. Cleavage of ring C by an retro-Diels-Alder mechanism led to A (*m/z *153) and B (*m/z *133) ions, was indicative of an unsubstituted A ring as well as two hydroxyl groups on B ring (Figure 2).

Comparison of the spectral and physical data with those published before, allowed us to establish the structures of the three other known compounds as quercetin 3-*O*-*β*-D-rutinoside (Q3Rut), myricetin 3-*O*-*β*-D-galactopyranoside (M3Gal), and quercetin 3-*O*-*β*-D-galactopyranoside (Q3Gal) (11-15). 

*Lymphocyte proliferation assay*

**Figure 3:**
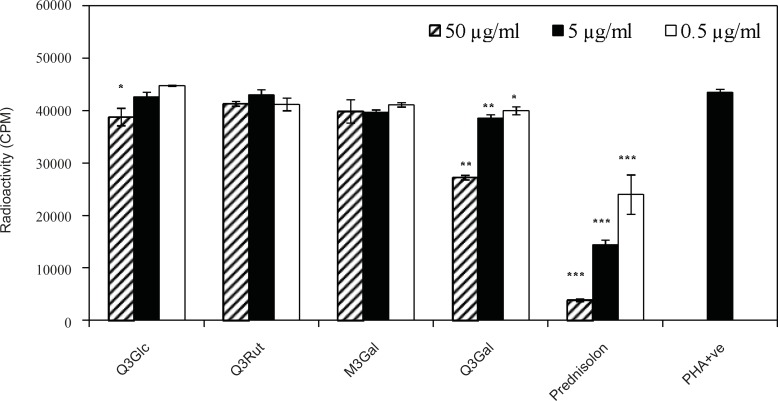
Effect of compounds 1-4 on the proliferation of T-cells. T-cells were stimulated with PHA in the presence of three different concentrations of quercetin 3-O-β-D-glucopyranoside (Q3Glc), quercetin 3-O-β-D-rutinoside (Q3Rut), myricetin 3-O-β-D-galactopyranoside (M3Gal), quercetin 3-O-β-D-galactopyranoside (Q3Gal) and prednisolon. Stimulated T-cells (PHA +ve) in the absence of compounds were used as controls. (* p < 0.05; ** p < 0.01; *** p< 0.001 vs PHA + ve control).

The anti-proliferation effect of the test compounds was determined by measuring the PHA-induced lymphocyte proliferation by determining radioactive thymidine incorporation. Comparisions of positive control in the absence of compounds and a standard drug were included to assess the activity of test compounds. T-cells were activated by phytohemagglutinin (PHA) mitogen in the presence of three concentrations (0.5, 5.0 and 50 μg/mL) of compounds 1-4 and prednisolone as a standard drug. As shown in Figure 3, weak inhibitory activity with IC_50_> 50 μg/mL was found for Q3Glc (1), and Q3Gal (4) with dose dependent (p-value > 0.05) suppression of lymphocyte proliferation by 10.7 ± 3.8 and 37.3 ± 0.5 %, respectively in the highest concentration (50 μg/mL), while Q3Rut (2) and M3Glc (3) were inactive in this dose. 

## Conclusion

Activated lymphocytes play a central role in the regulation of immunity, either directly as cytotoxic effectors or provide help for other cells that are important in the effector phase of the immune response (16). These data showed that lymphocyte suppression activity of flavonoids (1-4) were comparatively lower than prednisolon as a standard drug. Moreover as it is clear in Figure 3, suppressive activity of flavonoids with hydroxyl groups at both 3′-and 4′-positions in their B-ring (Q3Gal) were more than those with 3′-, 4′-and 5′-hydroxyl substitution (M3Gal).

 In addition, Q3Rut (2) and M3Glc (3) were inactive even in the highest concentration (50 μg/mL) which was agreed with other data that showed reducing *in vitro *biological activity of flavonoids in their glycosylation form (17). Although glycosylation of quercetin has reduced its *in-vitro *biological activity when compared to the corresponding aglycon form (17), but comparision of the lymphocyte antiproliferative activity of Q3Rut with Q3Glc and Q3Gal suggested that the type or size of sugar may also influence on T-cell suppressive activity. These results are agreed with other data about antiproliferative activities of quercetin derivatives such as Q3Glc, and rutin compared using different cancer cell lines. In different five cell lines, Q3Glc showed the most growth inhibition, while rutin showed the least potency (18). Concludingly, flavonoids as antioxidants have the ability to inhibit the superoxide producing enzymes like xanthine oxidase and protein kinase C (5). PKC plays an important role, as an early event, in the T-cell activation (6, 19); Therefore, the inhibition of PKC could be suggested as a mechanism of lymphocyte antiproliferative activity of the isolated flavonoids.
